# AMH in PCOS and Beyond—Rare Case Series

**DOI:** 10.3390/diagnostics16010123

**Published:** 2026-01-01

**Authors:** Ralitsa Robeva, Tzvetozar Mehandjiev, Roumen Dimitrov, Yuri Hranov, Silvia Andonova, Mihaela Mladenova, Atanaska Elenkova, George Hadjidekov, Sabina Zacharieva

**Affiliations:** 1 Department of Endocrinology, Faculty of Medicine, Medical University of Sofia, 1431 Sofia, Bulgaria; aelenkova@medfac.mu-sofia.bg; 2 USHATE “Acad. Iv. Penchev”, 1431 Sofia, Bulgaria; zacharieva67@gmail.com; 3 In Vitro Gynecological Medical Center “Dimitrov”, 1750 Sofia, Bulgaria; tzvetozar2@gmail.com; 4 Department of Obstetrics and Gynecology, University Hospital of Obstetrics and Gynecology “Maichin dom”, Faculty of Medicine, Medical University of Sofia, 1431 Sofia, Bulgaria; roumendim@abv.bg (R.D.); yurihranov61@gmail.com (Y.H.); 5 Genetic Medico-Diagnostic Laboratory “Genica”, 1463 Sofia, Bulgaria; sandonova@netscape.net (S.A.); mihaela.mladenova@gmail.com (M.M.); 6 IMDL “Genome Center Bulgaria”, 1612 Sofia, Bulgaria; 7 Department of Radiology, University Hospital “Lozenetz”, 1407 Sofia, Bulgaria; jordiman76@yahoo.com; 8 Department of Physics, Biophysics and Radiology, Medical Faculty, Sofia University “St. Kliment Ohridski”, 1504 Sofia, Bulgaria

**Keywords:** AMH, PCOS, supernumerary ovaries, GCT

## Abstract

**Background and Clinical Significance**: Anti-Müllerian hormone (AMH) is a dimeric glycoprotein secreted from the granulosa cells of the preantral and small antral follicles, which has entered routine clinical practice as a valuable tool for the diagnosis of different ovarian disorders. Increased AMH levels have been recommended as a criterion for polycystic ovary syndrome (PCOS). However, its widespread use remains limited due to analytical diversity and contradictory age-specific thresholds, among other factors that modulate AMH levels. **Case Presentation**: Herein, we present a rare case series of women with increased AMH levels. The difficulties in the differential diagnosis of patients with elevated AMH levels, because of PCOS combined with pituitary dysfunction, increased ovarian volume, or granulosa cell tumors (GCTs), are discussed. **Conclusions**: The presented rare cases of increased AMH emphasize the important role of AMH as a diagnostic marker in women with hypogonadotropic hypogonadism and granulosa cell tumors. On the other hand, it is still unknown if increased AMH produced by unusually enlarged or supernumerary ovaries should be considered as actual PCOS cases or as a specific subgroup. Additionally, the unusual case of GCTs with pronounced AMH and LH increase but normal steroids supports the pathophysiological role of AMH for the development of neuroendocrine dysfunction. Moreover, it suggests that GCTs should be considered in the differential diagnosis of chronic anovulation even in women with normal ovarian steroid production in case of unusually high AMH levels for the age. Further studies are needed to explain PCOS heterogeneity and to ensure proper differential diagnosis for every affected woman.

## 1. Introduction

Anti-Müllerian hormone (AMH) is a dimeric glycoprotein secreted from the granulosa cells (GCs), which is widely used as a diagnostic tool in different ovarian disorders [1,2,3]. AMH is produced mainly by the preantral and small antral follicles in the early follicle-stimulating hormone (FSH) during independent stages of development, while its expression decreases in the later FSH-dependent folliculogenesis [4]. Its functions in rodents are related to inhibition of the initial follicular recruitment and to the prevention of early ovarian reserve exhaustion; however, in humans, opposite results have also been reported, necessitating further research [4,5]. FSH and possibly androgens stimulate AMH synthesis in the GCs during early follicular development, but further increased AMH might partially limit FSH-induced follicular growth and aromatase activity. However, a rising estradiol/androgen ratio in the growing follicles inhibits AMH secretion, allowing for an increased capacity of aromatase activity and enhanced estradiol synthesis in the large antral follicles [6]. Unlike FSH, the luteinizing hormone (LH) does not alter AMH expression in the GCs of healthy women or normo-ovulatory patients with polycystic ovary syndrome (PCOS), but increases AMH in the GCs of PCOS individuals with chronic anovulation [7]. On the other hand, different oocyte-derived, e.g., bone morphogenic protein-15 and metabolic factors, might be important modulators of the ovarian AMH production [5].

More than 20 years ago, significantly increased AMH levels were found in women with polycystic ovary syndrome (PCOS), and were suggested as a surrogate marker for polycystic ovaries [8,9,10]. AMH overproduction decimates aromatase activity and enhances hyperandrogenicity and follicular maturation arrest in PCOS [6]. On the other hand, increased androgens suppress the negative feedback of steroids on the hypothalamus, thus, enhancing GnRH pulsatility and LH increase [8]. High LH levels stimulate additional androgen and AMH production in PCOS, and provoke the development of a vicious circle maintaining hormonal abnormalities and clinical symptoms [6,7,8]. Therefore, in 2023, AMH was included as a diagnostic criterium for PCOS in the international guidelines [3]. AMH levels in adults might suggest polycystic ovarian morphology with a high sensitivity and specificity, though they could not be used as a single criterium for a heterogeneous condition as PCOS [11]. However, the wide use of AMH is still limited because of analytic diversity, missing or contradictory age- and ethnic-specific thresholds, as well as additional factors modulating AMH levels, including obesity, hormonal drug use, and ovarian surgery [4,11,12,13,14].

Additionally, increased AMH in rare cases might suggest granulosa cell hyperplasia (GCH) or granulosa cell tumors (GCTs) [15], but the elevated AMH values often overlap in patients with PCOS and GCTs [16]. A few case reports have shown PCOS misdiagnosis in patients with a GCT [16,17], as well as the late diagnosis of a GCT due to atypical presentation and no visible ovarian tumor [18].

Herein, we describe four female patients presented with increased AMH levels due to increased ovarian volume, GCTs, and PCOS, complicated with pituitary dysfunction, and we discuss the difficulties in the evaluation of patients with increased AMH levels in real clinical practice as well as the role of AMH in the differential diagnosis and pathophysiology of PCOS. All patients gave their informed consent, and the study was approved by the Local Ethics Committee.

## 2. Case Series

### 2.1. Case 1

A 27-year-old Caucasian (Bulgarian) female (body mass index/BMI/25.7 kg/m^2^) consulted with an endocrinologist because of PCOS, diagnosed in adolescence based on hirsutism, menstrual disturbances, increased androgens, and luteinizing hormone (LH) levels (14.5 IU/L), as well as an ultrasound finding of polycystic ovaries. The patient had achieved the predicted normal height and no other hormonal disturbances had been found. Thus, therapy with oral contraceptives had been started for eight years. After cessation of the pills, the amenorrhea persisted, despite normal androgens. A gestagen test was negative, while AMH levels were significantly increased. Hormonal investigations in a tertiary Endocrine Department (USHATE “Acad. Iv. Penchev”, Sofia, Bulgaria) revealed hypogonadotropic hypogonadism with decreased estradiol and low gonadotropins (Table 1). The patient did not follow a strict diet plan and was not engaged in vigorous physical activity. Additionally, hyposomatotropism was found, while thyroid, prolactin, and cortisol secretion patterns were unaffected. Pituitary imaging showed two microadenomas (both with diameter 4 mm). Hormone replacement therapy was started and regular hormonal, visual field, and imaging follow-up was recommended. Additionally, the patient was with impaired glucose tolerance and severe hyperinsulinemia (HOMA-IR 7.11); thus, metformin treatment was also prescribed.

### 2.2. Case 2

**Figure 1:**
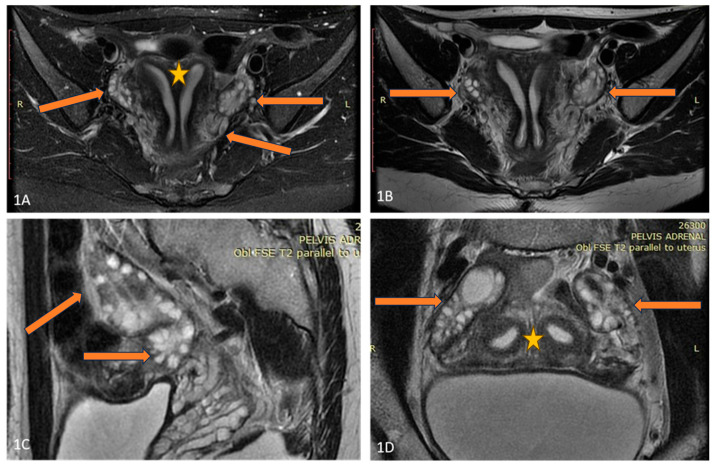
(**A**–**D**) Axial T2FSE Fat Sat and T2FSE images (**A**,**B**) of the patient demonstrate uterus didelphys (orange star) and double bilateral ovaries (arrows). A sagittal T2 FSE image (**C**) shows double, partially fused ovaries. Two separate uterine cavities and bilateral ovaries are seen in the paracoronal oblique plane, parallel to the uterine axis (**D**).

A 25-year-old lean Caucasian woman (BMI 19.0 kg/m^2^) was referred to the Endocrine Department because of hirsutism. After menarche at the age of 16 years, she had suffered from oligomenorrhea. Oral contraceptive treatment had been provided, but stopped because of the development of breast fibroadenoma. However, over the last three years, the menstrual pattern has become regular. She complained of slightly increased non-progressive hair growth on androgen-dependent zones since puberty, which was treated locally by a dermatologist. Polycystic ovary syndrome was suspected, based on her complaints (Table 1). Laboratory results showed upper-normal testosterone and increased levels of 17-OH progesterone. Therefore, congenital adrenal hyperplasia was excluded through a short Synacthen test. The blood count, blood glucose, lipid panel, and routine biochemical parameters, as well as thyroid function, were normal, while the AMH was significantly increased (23 ng/mL). A pelvic ultrasound revealed uterus didelphys with two separated cervices and a septated distal vagina. The ovaries were significantly increased in volume (“double ovaries”) but without pathological findings. The subsequent magnetic resonance imaging (MRI) (Figure 1) confirmed the ultrasound description. No tumor was found in the ovaries, and the investigated tumor markers (CEA, hCG, alpha-fetoprotein, LDH, and Ca-125) were in the reference ranges.

An abdominal ultrasound did not show any abnormalities, while a QF-PCR genetic test revealed the presence of two X-chromosomes. She had no concomitant diseases except a nodose euthyroid struma grade 1B. Observation and AMH follow-up was recommended to the patient. One year later, she conceived spontaneously and gave birth to a healthy baby. Eight years later the patient still has no gynecological complaints. Her AMH level decreased, but remained elevated for the age (13.4 ng/mL).

### 2.3. Case 3

A 33-year-old overweight Caucasian female patient (BMI 27.0 kg/m^2^) consulted with an endocrinologist and gynecologist because of recurrent episodes of abnormal uterine bleeding. Several years after menarche (at the age of 16 years), regular periods were experienced, but since the age of 26 she complained of frequent menometrorrhagia leading to mild anemia. Thyroid dysfunction, hyperprolactinemia, and congenital adrenal hyperplasia were excluded by appropriate tests. The patient complained of mild hirsutism and acne since puberty. Liver and renal function as well as estradiol levels were normal. The androgen and LH levels were significantly increased by multiple investigations. Additionally, elevated AMH levels for her age (Table 1) were found.

**Figure 2:**
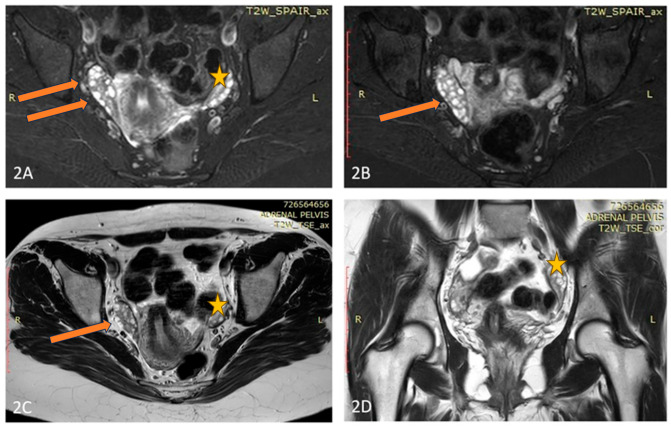
(**A**–**D**) Axial T2 Fat Sat (**A**,**B**), Axial T2 (**C**), and Coronal T2 (**D**) images of the patient demonstrate an elongated, fused double right-sided ovary (arrow), and normal size and appearance of the left ovary (star).

A pelvic ultrasound showed an increased size of the right ovary without any evidences of tumor mass. An MRI of the abdomen and pelvis also did not reveal any suspicious formation but proved the doubled size of the right ovary compared to the left one (Figure 2). Tumor markers were negative. Because of abnormal uterine bleeding, a therapy with oral contraceptives was introduced with a rapid alleviation of symptoms. No substantial decrease of AMH levels were found under hormonal therapy. An MRI of the pituitary showed a 6 mm-large pituitary microadenoma, without overt hormonal secretion. Additionally, a cytogenetic study on a heparin-blood sample was performed, showing a karyotype 45,XX,der(14;21)(q10;q10) with balanced Robertsonian translocation. The patient was referred for genetic counselling, where possible reproductive risks were explained. A segregation analysis in the family revealed the paternal origin of the translocation between chromosomes 14 and 21. The patient conceived on the first cycle of ovulation induction. Unfortunately, after amniocentesis was performed, a fetal trisomy 21 with mosaic ratio 14% was detected and the family decided to terminate the pregnancy. Thereafter, the dysfunctional uterine bleeding episodes persisted, as well as the chronic anovulation.

### 2.4. Case 4

**Figure 3:**
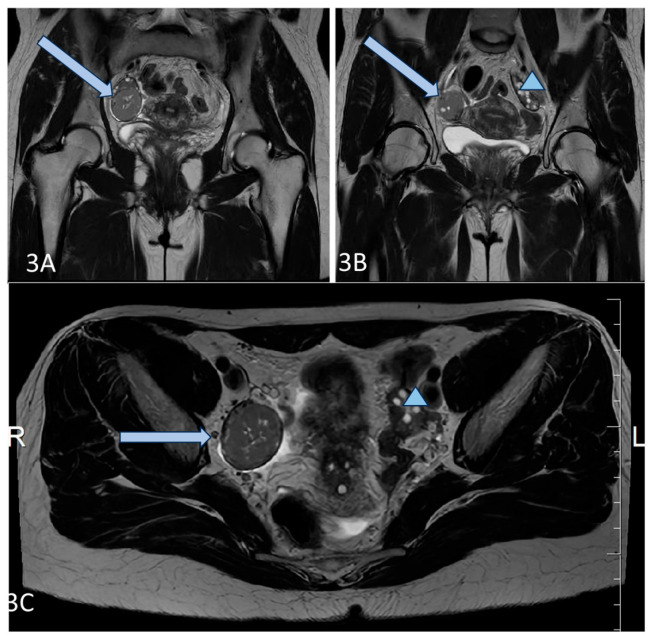
(**A**–**C**) Axial T2 (**A**,**B**), and Coronal T2 (**C**) images of the patient demonstrate a well-circumscribed mass, representing a solid granulosa cell tumor at the level of the right ovary (blue arrow), and normal size and appearance of the left ovary (blue triangle).

A 36-year-old lean Caucasian female patient (BMI 22.6 kg/m^2^) with PCOS was referred to the Endocrinology Department. The patient complained of mild hirsutism and oligomenorrhea since menarche (age 17 years). Despite chronic anovulation, she conceived spontaneously several times (two miscarriages and two live births). Increased LH and AMH levels (varying up to 50 ng/mL by unspecified analyses) as well as ultrasound evidence for polycystic ovaries were found by secondary care evaluation, and therapy with oral contraceptives was started. The patient had regular menstrual bleeding on therapy, but decided to stop the pill, leading to continuous amenorrhea, attributed to her PCOS, for several years. In 2021, the investigations in the Endocrinology Department showed normal testosterone and estradiol levels, increased LH and AMH (Table 1), as well as normal tumor markers (Ca-125, HE4). A pelvic ultrasound by an experienced gynecologist suggested 2/2 cm formation of the right ovary and no pathological findings of the left ovary. Magnetic resonance imaging (MRI) and laparoscopy were recommended because of a suspected fibroma/granulosa cell tumor, but the patient refused further investigations. Two years later, she performed the recommended MRI and a solid tumor 42/38/35 mm was described (Figure 3). She repeated her hormonal tests because of persisting amenorrhea, and again increased LH and AMH levels and normal androgens were detected (Table 1).

The patient agreed to an operation, and a right ovariectomy was performed in the University Hospital of Obstetrics and Gynecology “Maichin dom”, Sofia, Bulgaria. A 5 cm large ovarian tumor was removed, and the histological evaluation confirmed the granulosa cell tumor—adult type. The patient was sent to an oncologist, but refused additional chemotherapy or surgery. One year after the operation, she had regular menstruation, and normal AMH and LH levels (Table 1).

## 3. Discussion

The present case series shows the important place of AMH in a PCOS diagnosis and a differential diagnosis. Our first patient (C1) was diagnosed with PCOS based on convincing hormonal and clinical symptoms in adolescence, and treated with oral contraceptives accordingly. After discontinuation of the pills, she was amenorrhoeic, normoandrogenic, and a negative gestagen test confirmed a hypoestrogenic state. Partial hypopituitarism was revealed based, most likely, on the developed pituitary adenomas, though another unknown acquired cause could not be excluded. However, the AMH levels were strongly increased corresponding to the underlying PCOS phenotype A. Most patients with congenital hypopituitarism show decreased or low-normal AMH levels, while patients with late-onset pituitary dysfunction are usually with normal hormonal concentrations [20,21]. AMH levels in women with functional hypothalamic amenorrhea (FHA) might be lower, similar, or higher compared to healthy controls [22,23]. The discrepancies emerge from the inclusion of two different FHA phenotypes determined by the presence of polycystic ovarian morphology (FHA ± PCOM). FHA + PCOM women show higher AMH levels and increased stimulated LH levels compared to FHA–PCOM, resembling PCOS [22,24]. According to Carmina et al., 10% of women with FHA could have a coexisting polycystic ovary syndrome [25]. On the other hand, the changing clinical features of PCOS women in cases of hypothalamic gonadotropin suppression might be viewed as “phenotypic conversion rather than ‘co-existence’” [24]. The differential diagnosis between PCOS phenotype D and FHA + PCOM might be difficult, considering the overlapping PCOM and menstrual disturbances by lacking a longitudinal follow-up [23,24]. Conversely, our patient shows a clear coexistence of hyperandrogenic PCOS phenotype A and subsequently developed hypogonadotropic hypogonadism, retaining significantly elevated AMH levels and pronounced insulin resistance. Currently, pituitary imaging is recommended in patients with FHA in cases of compressive symptoms or pituitary hormone alterations [26]. Unusually high AMH in women with presumable FHA or partial hypopituitarism might be another indication for an MRI exclusion of a pituitary lesion.

Our next three patients presented with approximately a 30% increase of AMH levels compared to the upper reference ranges for PCOS women from the same ethnic group [19].

The second (C2) patient showed strongly increased AMH compared to usual AMH levels, and the imaging studies revealed uterus didelphys and a significantly increased ovarian mass (“doubled” ovaries by MRI)**.** She also complained of mild hirsutism despite normal testosterone levels and regular ovulatory function. Usually, strongly increased AMH levels have been associated with the more severe PCOS phenotype A [27]. Therefore, a cryptic GCT had been initially suspected, but no tumor mass was found. The successful pregnancy and the decrease of AMH by follow-up years later suggested a benign condition. The patient fulfilled the PCOS criteria for phenotype C (hirsutism and increased ovarian volume), though a specific condition associated with the unusually large ovarian mass could not be excluded. The associations between AMH and Müllerian duct abnormalities in PCOS is poorly investigated. One large Asian study explored the prevalence of uterine variations in PCOS women with low and high AMH levels and found ten-times increased prevalence of a unicornuate uterus in high-AMH individuals compared to others; however, uterus didelphys was too rare in both groups to draw any conclusions [28]. Further large studies are needed to find the possible associations between the early prenatal AMH increase, Müllerian duct abnormalities, and ovarian dysfunction later in life.

Our third patient (C3) showed chronic anovulation and mild hirsutism as PCOS features, along with ovarian asymmetry and an increased right ovarian mass. A GCT was also suspected because of the later onset of complaints after a long period of regular menstruation patterns and increased concentrations of AMH for her age. The extensive investigations did not reveal a tumor mass. Nevertheless, follow-up was recommended, because it may take up to 8 years for a microscopic tumor secreting AMH to be detected by imaging studies [15].

Considering the Robertsonian translocation (RT) found in the patient, it is interesting to explore if her complaints, asymmetric ovaries, and increased AMH levels might be associated with chromosome aberration. Robertsonian translocation (RT) represents a fusion of two acrocentric chromosomes occurring in 1 of every 800 newborns [29]. Usually, adult RT carriers are healthy, but carry the increased risk of some malignancies [30]. On the other hand, 60% of adult granulosa cell tumors have chromosomal imbalances, e.g., losses of 22 q (31%), and trisomy 14 (25%) [31]. Akbulut et al. have reported a patient with a simple virilizing form of congenital adrenal hyperplasia, triple translocation [t(9;11;12)], and a giant ovarian granulosa cell tumor [32]. On the other hand, recently, a woman with PCOS and balanced reciprocal translocation has been described [33]. Further studies are needed to reveal if RT carriers are at an increased risk of PCOS, granulosa cell hyperplasia, or ovarian tumors.

Our fourth patient (C4) had been diagnosed and treated for PCOS for several years because of chronic anovulation, an increased LH/FSH ratio, and mild hirsutism. However, her testosterone, estradiol, and DHEAS levels were normal, while her AMH levels were significantly higher for her age. A GCT was detected by imaging studies, thus, confirming the important role of AMH as a tumor marker. Moreover, the decrease of AMH levels after surgery was associated with rapid LH normalization, FSH and estradiol increase, as well as a regular menstruation pattern, suggesting the pathological influence of high AMH levels on the pituitary and the LH/FSH ratio, respectively.

Similarly, Chi et al. described a hyperandrogenic Chinese woman previously diagnosed with PCOS and treated for infertility for several years, who underwent additional extensive investigations because of unusually increased AMH levels. The following MRI, laparoscopy, and histological evaluations proved the presence of a GCT [34]. Similarly, our patient had been treated for several years for PCOS until the tumor mass became visible by ultrasound evaluation. However, the GCT in our patient did not produce testosterone or estradiol, conversely to the GCTs described in the literature [34,35,36]. Thus, the increased LH secretion could not be explained by the elevated testosterone levels, as suggested in the other cases [36], and might be directly related to the central effects of the pronounced AMH increase.

The similar clinical and hormonal characteristics of PCOS and an early-stage GCT in reproductive-aged women could originate from the common pathophysiological pathways associated with AMH signaling [34]. In experimental female animals, AMH induced a significant increase of gonadotropin releasing hormone (GnRH) secretion and LH by stimulating a subpopulation of GnRH neurons expressing the AMH-receptor [37]. An application of AMH in pregnant mice induced neuroendocrine dysfunction with increased LH pulsatility and hyperandrogenism persisting in the exposed female’s offspring [38]. Thus, the increased AMH levels resulting from increased AMH secretion in the granulosa cells, an increased number of granulosa cells (“mass effect”), or granulosa cell tumors with moderate AMH secretion might affect the gonadotropin ratio and steroidogenesis, leading to similar clinical symptoms [39].

Currently, AMH might be used for the diagnosis of PCOS in adults, but not adolescents, as a marker for polycystic morphology in accordance with the accepted diagnostic algorithms [3]. Recently, a cut-off of 3.2 ng/mL has been validated in a large Finnish cohort as an accurate tool for detecting polycystic ovaries that could be used widely in clinical practice [40]. Conversely, no AMH cut-off could distinguish polycystic ovaries from GCTs. Our case report shows that strongly increased AMH might be a sole marker for GCTs in women. Thus, a GCT should be considered in the differential diagnosis of chronic anovulation even in women with normal ovarian steroid production in cases of unusually high AMH levels for the age of the patient.

## 4. Conclusions

Our case series present rare cases of increased AMH in patients with PCOS and other conditions, emphasizing the important role of AMH as a diagnostic marker in women with hypogonadotropic hypogonadism and granulosa cell tumors. On the other hand, the described cases raise the question of whether women with increased AMH due to unusually enlarged or supernumerary ovaries should be considered as true PCOS cases or as a specific subgroup. Additionally, the unusual case of GCTs with pronounced AMH and LH increase but normal steroids indirectly supports the pathophysiological role of AMH for the development of neuroendocrine dysfunction. The proper evaluation of very high AMH is of great clinical importance, considering its ever-expanding use in PCOS diagnostics. Further studies are needed to explain PCOS heterogeneity and to ensure a precise differential diagnosis, thus providing individualized management for every affected woman.

## Figures and Tables

**Table 1:** Hormonal levels of the investigated patients: case 1 (C1), case 2 (C2), case 3 (C3), and case 4 (C4). LH—luteinizing hormone; FSH—follicle-stimulating hormone; TT—total testosterone; DHEAS—dehydroepiandrosterone; AMH—anti-Müllerian hormone; OP—surgical tumor extirpation; * Testosterone measured by different kit with an upper referent range (<1.7 mmol/L). ^ the samples were obtained in the follicular phase of the menstrual cycle for patients C2, C3, and C4—post-OP, and in amenorrhea for C1 and C4—pre OP1/2. ** Upper range (97.5 percentile) of AMH (ELISA, Demeditec Diagnostics GmbH, Kiel, Germany) for the same ethnic group is 5.45 ng/mL for healthy women in the reproductive age and 12.16 ng/mL for women with PCOS [19].

Hormone:	LH ^	FSH	Prol	TT	E2	DHEAS	AMH **	Inhibin B
Referent ranges	2–10 IU/L	1–10 IU/L	<600 mIU/L	<3.5 nmol/L	90–550 pmol/L	0.8–9.0 µmol/L	1.18–6.8 ng/mL	9–33 pg/mL
C1	2.42	1.2	235	1.32*	24	10.44	14.9	
C2	4.7	7.2	372	3.3		7.1	23	
C3—basic	14.6	5.83	92	4.6		3.3	16.9	
C3—follow up	19.7	6.4	127	2.0 *	167	3.3	17.0	
C4—pre-OP1	29.6	2.3	257	2.8	105	6.3	11.9	
C4—pre-OP2	31.6		151	1.0 *	79	7.3	17.1	45
C4—post-OP	6.9	8.4	267	1.3 *	321	7.8	2.24	22

## Data Availability

The original contributions presented in this study are included in the article. Further inquiries can be directed to the corresponding author.
